# Whole Transcriptome Analysis: Implication to Estrous Cycle Regulation

**DOI:** 10.3390/biology10060464

**Published:** 2021-05-25

**Authors:** Xiaopeng An, Yue Zhang, Fu Li, Zhanhang Wang, Shaohua Yang, Binyun Cao

**Affiliations:** College of Animal Science and Technology, Northwest A&F University, Xianyang 712100, China; anxiaopengdky@163.com (X.A.); zhang_yue_0624@163.com (Y.Z.); lfu558389@gmail.com (F.L.); zhanhangwang12@gmail.com (Z.W.); yangshaohua63@gmail.com (S.Y.)

**Keywords:** circular RNA, microRNA, long non-coding RNA, messenger RNA, estrous cycle

## Abstract

**Simple Summary:**

The databases of mRNA and non-coding-RNAs (miRNA, circRNA, lncRNA) in the ovary of Xinong Sannen goat were reported in this study. The differential expression of mRNA and non-coding RNAs were analyzed, and the comprehensive analysis of the four databases provided RNA networks that regulate estrous cycle, which is essential to improve reproduction.

**Abstract:**

Estrous cycle is one of the placental mammal characteristics after sexual maturity, including estrus stage (ES) and diestrus stage (DS). Estrous cycle is important in female physiology and its disorder may lead to diseases, such as polycystic ovary syndrome, ovarian carcinoma, anxiety, and epilepsy. In the latest years, effects of non-coding RNAs and messenger RNA (mRNA) on estrous cycle have started to arouse much concern, however, a whole transcriptome analysis among non-coding RNAs and mRNA has not been reported. Here, we report a whole transcriptome analysis of goat ovary in estrus and diestrus periods. Estrus synchronization was conducted to induce the estrus phase and on day 32, the goats shifted into the diestrus stage. The ovary RNA of estrus and diestrus stages was respectively collected to perform RNA-sequencing. Then, the circular RNA (circRNA), microRNA (miRNA), long non-coding RNA (lncRNA), and mRNA databases of goat ovary were acquired, and the differential expressions between estrus and diestrus stages were screened to construct circRNA-miRNA-mRNA/lncRNA and lncRNA-miRNA/mRNA networks, thus providing potential pathways that are involved in the regulation of estrous cycle. Differentially expressed mRNAs, such as *MMP9*, *TIMP1*, *3BHSD,* and *PTGIS*, and differentially expressed miRNAs that play key roles in the regulation of estrous cycle, such as miR-21-3p, miR-202-3p, and miR-223-3p, were extracted from the network. Our data provided the miRNA, circRNA, lncRNA, and mRNA databases of goat ovary and each differentially expressed profile between ES and DS. Networks among differentially expressed miRNAs, circRNAs, lncRNAs, and mRNAs were constructed to provide valuable resources for the study of estrous cycle and related diseases.

## 1. Introduction

Estrous cycle is one of the physiological characteristics of placental mammals induced by sex hormones after sexual maturity, including estrus stage (ES) and diestrus stage (DS). The length of time between two consecutive ovulation periods is defined as an estrous cycle. The same phenomenon in humans is called menstrual cycle, which is differentiated by the pattern of the endometrium being removed when the mammals are not pregnant after the cycle. In humans, the endometrium would be eliminated from the body at the menstrual period, while it would be absorbed in non-primate mammals [1]. Both menstrual and estrous cycles are closely related to complex hormonal intercommunications of the hypothalamic-pituitary-ovarian axis [2,3,4].

In goat, the reproduction is spontaneously ovulating and seasonally polyestrous, with the length of estrous cycle being from 18 to 24 days [5]. Goats are sexually active only in the ES, and humans can keep sexually active at any time, even not in the ovulatory period. The onset and duration of estrus in goat is related to various factors, such as hormone, photoperiod, age, climate, and food supply. Generally, in temperate areas, goats are found to breed in the fall and winter due to the annual variations of photoperiods. In tropical areas, however, goats are considered to be in regular estrous cycles throughout the year [5]. Nevertheless, the structure of the reproductive system and the release of sex hormones are similar between human and goat. Studies in rodents show that disorder of estrous cycle participates in disease progression, such as ovarian carcinoma [6], anxiety [7,8], and epilepsy [9]. Recently, it was reported that neuronal chromatin organization fluctuates with the estrous cycle in the brain [10], and rats in diestrus stage are easier to display memory impairment than those in estrus stage when they undergo restraint and social isolation stress [11], indicating an importance of estrous cycle in physiological regulation. Disorder of estrous cycle is a dramatic symptom of polycystic ovary syndrome (PCOS), which disturbs adolescent females in reproductive ages [12]. The regulation of estrous cycle in goat breeding is also important for milk and meat industries to meet the year-round demands of consumers [1]. In the latest years, effects of non-coding RNAs and messenger RNA (mRNA) on estrous cycle have started to arouse much concern [13,14,15,16,17], however, joint transcriptome analysis among non-coding RNAs and mRNA has not been reported.

Non-coding RNAs consist of microRNA (miRNA), circular RNA (circRNA), and long non-coding RNA (lncRNA). MiRNAs are a class of endogenous non-coding RNAs at a length of about 20 nucleotides, regulating gene expression by promoting mRNA degradation or preventing translation [18,19]; CircRNAs, derived by non-classical alternative splicing, are another class of endogenous non-coding RNAs that can expropriate miRNAs as a sponge to block miRNAs from binding to target genes [20,21,22]. LncRNAs are a category of transcripts longer than 200 nucleotides without open reading frames that achieve functions through interacting with DNA, other RNAs, and proteins by base complementation or secondary structure generated by RNA folding [23]. The mRNA expression profile of ovary between uniparous and multiparous Anhui white goats [24], the miRNA expression in Chuanzhong black goat ovarian stroma and follicles [25], circRNA expression difference in pre-ovulatory ovarian follicles between Boer goat and Macheng black goat [26], and lncRNA expression difference in ovary of Anhui white goat at different estrous cycle periods [14], have been investigated by sequencing in previous studies, which provide resources for studies on goat fertility. Moreover, the mRNA and lncRNA databases of goat ovary in Chuanzhong black goat between high- and low-fecundity goats have also been analyzed [27]. However, the coalition analysis between each database is not reported in goat. An integrated analysis of lncRNA, miRNA, and mRNA in ovary at different phases of estrous cycle has been reported to explore the regulation networks in large white sows, and it reveals a novel insight in the regulation of pig fertility [28]. In this study, the databases of miRNA, circRNA, lncRNA, and mRNA in the ovary of Xinong Sannen goat in ES and DS were acquired and analyzed in combination. The differentially expressed ones were extracted to construct circRNA-miRNA-mRNA/lncRNA and lncRNA-miRNA/mRNA networks that participated in estrous cycle, therefore contributing to further investigation in the comprehensive regulation of estrous cycle.

## 2. Methods

### 2.1. Animal and Ethics

Two-year-old female Xinong Sannen goats were kept in a breeding basement of Northwest A&F University with enough space. Feed and water consumption was ad libitum. Female multiparous healthy goats weighing around 60 kg were selected for this experiment. This experiment was conducted in summer, and the goats were in the anestrus stage. Estrus synchronization was conducted with PGF2α, medroxyprogesterone, FSH, and PMSG to induce the estrus stage of goats. At the first day, 0.2 mg of PGF2α was given by intramuscular injection and a vaginal medroxyprogesterone suppository was applied. On day 10, 20 IU of FSH was dosed twice in an 11 h interval by intramuscular injections. On day 11, the vaginal sponge was removed, and 200 IU of PMSG and 0.1 mg of PGF2α were injected intramuscularly. On day 12, male goats were used to differentiate estrus from diestrus animals (to avoid breeding, the abdomen of the ram is tied with a white cotton cloth that has ropes on the four corners during the test), and females that accepted males to climb across were regarded as in ES. On day 32, the female goats were reluctant to the climb of males, which shows them to be in DS. Three random goats in estrus or diestrus were slaughtered respectively after anesthetization, and ovary tissues were frozen in liquid nitrogen immediately. RNAiso Plus (Takara, Tokyo, Japan) was applied to isolate total RNA of tissues in accordance with manufacturer’s protocol. All of the procedures were approved by the Animal Care and Use Committee of the Northwest A&F University and conformed to national guidelines (ethic code: #0726/2018).

### 2.2. miRNA Sequencing

Total RNA was separated by size using agarose gel electrophoresis for segments of 18–30 nucleotides to be linked with 3′adaptors. The products were purified further to get segments of 36–44 nucleotides by Urea-PAGE gel, and linked with 5′adaptors to get miRNA samples. Then, reverse-transcription PCR was conducted, whose products went through a separation by 3.5% agarose gel to get segments of 140–160 base pairs. The gel extraction product was prepared as miRNA library for miRNA sequencing. After sequencing, the reads of low quality were filtered, and adapters were removed to obtain tag sequences of small RNA. The tags were then annotated, and miRNAs that existed in the miRBase database (http://www.mirbase.org/ (10 March 2021)) were identified. Novel miRNAs were identified by the prediction of hairpin structure. The expression profile of miRNA in Xinong Sannen goat ovary was obtained and differentially expressed miRNAs were screened. The target genes of differentially expressed miRNAs were predicted, and gene ontology enrichment (http://geneontology.org/ (10 March 2021)) and KEGG pathway enrichment (https://www.genome.jp/kegg/ (10 March 2021)) of predicted target genes were performed to analyze the possible function of differentially expressed miRNAs.

### 2.3. circRNA Sequencing

The ribosomal RNA was removed from total RNA and linear RNA was degraded by Rnase R. Then, fragmentation buffer was applied to obtain short fragments of circRNAs, which were used as templates for first-strand cDNA synthesis by random hexamers. Second-strand cDNA was synthesized by dNTPs, RNase H, and DNA polymerase I. A QiaQuick PCR kit was used to purify the products with EB buffer. After end repair, base-A addition, and sequencing adaptor addition, the products were purified again by size with an agarose gel. PCR amplification was conducted to establish a circRNA library for circRNA sequencing by Illumina HiSeq 2500. The raw data acquired by sequencing (three animals from each category) were filtered to get the high-quality clean reads, which were compared with the reference genome. Both ends of the unmapped reads were intercepted as anchors reads. The circRNAs were identified by Find_circ software, and the information, such as type, distribution, expression, and predicted target relation, was analyzed and summarized.

### 2.4. mRNA and lncRNA Sequencing

The ribosome RNA of total RNA was removed with the remaining mRNA to reserve all non-coding RNAs as much as possible. The obtained mRNA was broken into short segments at the length of 200–500 nucleotides as templates for cDNA synthesis after ribosomal RNA. First-strand cDNA was synthesized by random hexamers and then dNTPs, RNase H, and DNA polymerase I were applied to acquire the second strand. PCR amplification was performed after ligate adapter and Uracil-N-glycosylase treatment to establish the library for sequencing. The quality of the data was assessed to remove the reads with adapter and N content greater than 10% and the low-quality reads (the number of bases with Q ≤ 20 accounts for more than 50% of the entire read), and then the data of high quality was used for analysis. To improve the efficiency of sequencing, the high-quality clean reads that mapped to the ribosome database were removed since ribosome RNA makes up >80% of cellular RNA and could obstruct the detection of other RNAs [29]. The filtered reads were mapped to the reference genome database to build the mRNA library and calculate the expression of mRNAs. The reads per kilobase transcriptome per million mapped reads (RPKM = entire exon reads/mapped reads in millions × exon length in kb) method was used to normalize the gene expression levels. RPKM > 1 was used as the threshold to judge gene expression. The significance limitation of the *p*-value in numerous tests was fixed on the basis of false discovery rate (FDR). Standardized gene expression levels of groups were measured using the fold changes (log_2_ |Fold Change|) by DESeq (version 1.18.0). Finally, the standards of (i) |log_2_ (Fold Change)| > 1 and (ii) *p* < 0.05 were utilized to determine the significance of gene expression differences. Enrichments of gene ontology (http://geneontology.org/ (10 March 2021)) and KEGG pathway (https://www.genome.jp/kegg/ (10 March 2021)) were performed to analyze the processes that differentially expressed mRNAs participated in. LncRNAs were differentiated from mRNA using CNCI and CPC software by the evaluation of coding ability.

## 3. Results

### 3.1. Overview of circRNA Sequencing

**Figure 1:**
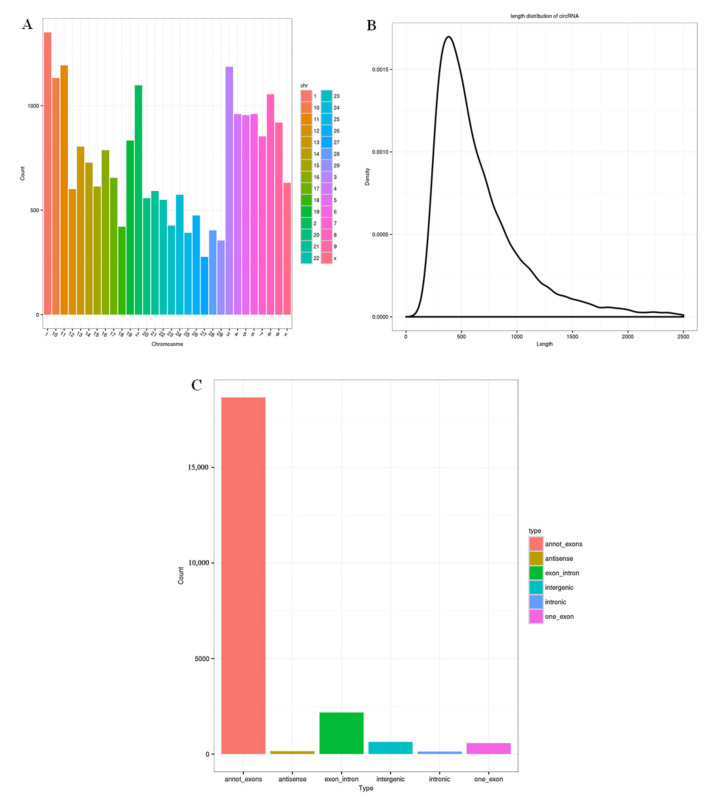
The location, length, and type of identified circRNAs. (**A**) The chromosome distribution of identified circRNAs, (**B**) the length distribution of identified circRNAs, and (**C**) the number of the six types of circRNA.

An average of 84,629,465 (DS) and 80,303,146 (ES) clean reads were obtained in two libraries. After the data were quality-controlled and filtered, 99.28% (DS) and 98.95% (ES) of high-quality (HQ) clean reads were generated. The mapped rRNA reads were removed from HQ clean reads, and then 20 base pairs at both ends of the unmapped reads were picked as Anchors Reads, which were later aligned to the caprine genome. In this experiment, 22,333 novel circRNAs were found from the mapped reads and no existing circRNAs were detected. The information of circRNAs is shown in Supplementary Table S1, including source gene ID, chromosome and genomic location, length, and annotation type. The distribution of the identified circRNAs on caprine chromosomes is presented in Figure 1A, which showed that most circRNAs were on chromosomes 1, 2, 3, 8, 10, and 11. In general, most circRNAs were 400 nucleotides in length (Figure 1B) and six types of circRNAs were identified, with annot_exon being the most common type (Figure 1C).

### 3.2. CircRNA Source Gene Analysis and Differentially Expressed circRNAs Analysis

**Figure 2:**
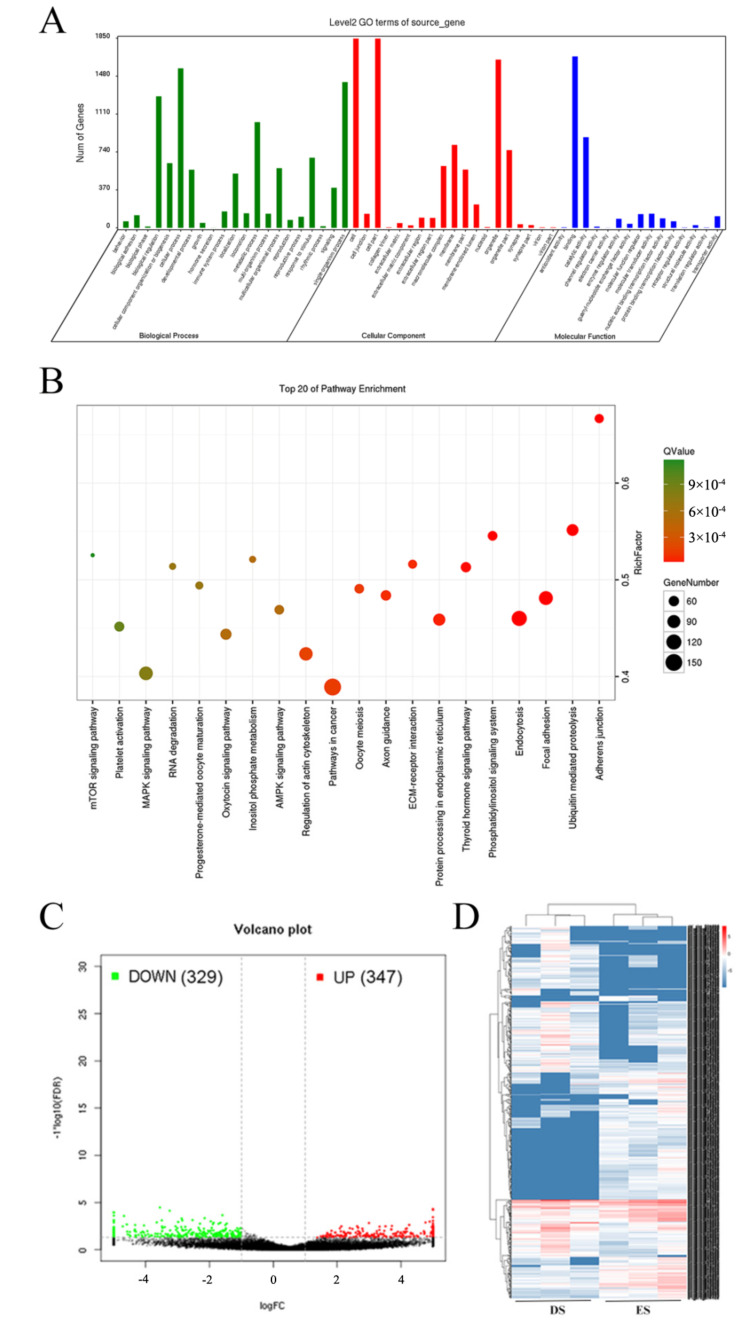
The analysis of circRNA source genes. (**A**) The enriched GO terms of circRNA source genes. GO enrichment of circRNA source genes was conducted to annotate the genes that were potentially spliced to circRNAs in goat ovary. Overall, 9566 of the source genes played roles in biological process, 3274 genes participated in cellular component, and 8786 genes were in molecular function. (**B**) The enriched KEGG pathways of circRNA source genes. CircRNA source genes were analyzed by the KEGG database, and 287 KEGG pathways that the source genes participated in were enriched. The top 20 pathways were selected to show in the figure. (**C**) The number of differentially expressed circRNAs between ES and DS groups. There were 347 circRNAs upregulated and 329 circRNAs downregulated in the ES group compared to the DS group (*p* < 0.05). (**D**) The heatmap of differentially expressed circRNAs.

GO terms of circRNA source genes were enriched in three aspects (Supplementary Table S2): Biological Process (9566 genes), Cellular Component (3274 genes), and Molecular Function (8786 genes). It was found that circRNA source genes are mainly involved in cellular process (GO:0009987), single-organism process (GO:0044699), biological regulation (GO:0065007), cell (GO:0005623), cell part (GO:0044464), organelle (GO:0043226), binding (GO:0005488), and catalytic activity (GO:0003824) terms (Figure 2A). The KEGG pathway database was applied to analyze metabolic processes that the source gene participated in, and 287 KEGG pathways were found (Supplementary Table S3). The top 20 enriched pathways are shown in Figure 2B, including Oocyte meiosis (ko04114), Thyroid hormone signaling pathway (ko04919), Oxytocin signaling pathway (ko04921), and Progesterone-mediated oocyte maturation (ko04914), which are closely related to reproduction traits.

When |log_2_ (Fold Change)| > 1 and *p* < 0.05, the circRNA would be regarded as differentially expressed circRNA. Overall, 676 differentially expressed circRNAs were discovered in the ES group compared to the DS group, including 347 upregulated and 329 downregulated ones (Figure 2C). The heatmap of cluster analysis is shown in Figure 2D. Besides, all acquired circRNAs were analyzed to predict the targeted relationship with existing miRNAs, and 22,207 circRNAs were found to be combined with 433 miRNAs, creating 447,870 combination opportunities between circRNAs and miRNAs (Supplementary Table S4).

### 3.3. Sequencing and Analysis of miRNA

**Figure 3:**
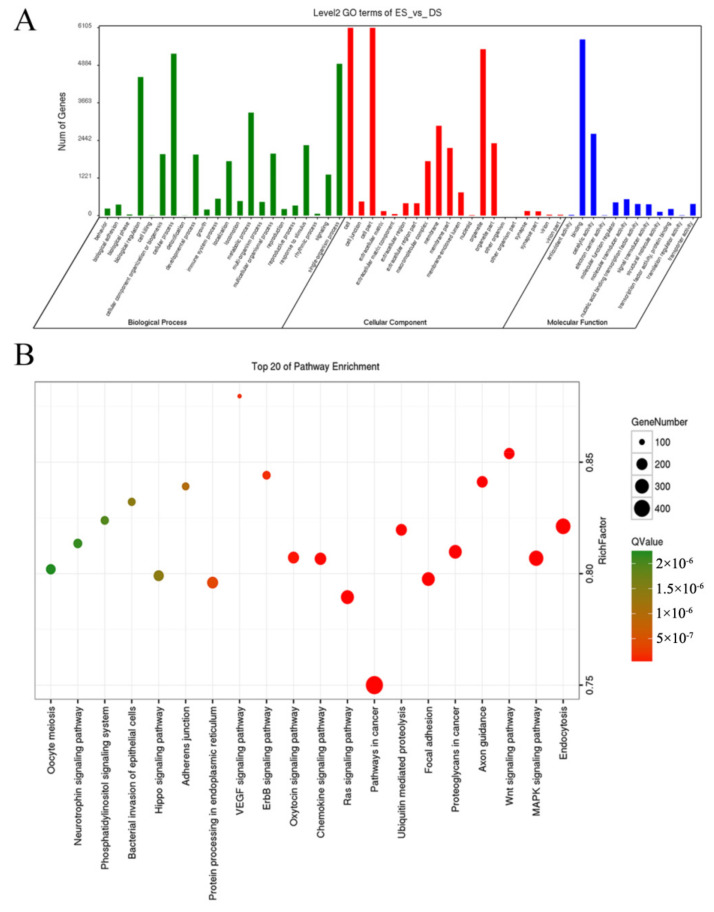
GO and KEGG enrichments of predicted target genes of differentially expressed miRNA. (**A**) The enriched GO terms of differentially expressed miRNA target genes, and (**B**) the enriched KEGG pathways of differentially expressed miRNA target genes. To assess the function of differentially expressed miRNA in different stages of estrous cycle, the target genes of the miRNAs were predicted, and GO enrichment (**A**) and KEGG enrichment (**B**) were conducted.

Low-quality reads (quality value less than 20 or containing N bases) were removed to obtain tag sequences, which were aligned with miRNAs in the miRBase database (http://www.mirbase.org (10 March 2021)) to identify existing or known miRNAs. Novel miRNAs were identified with hairpin motif prediction referring to reference sequences. The expression of miRNAs is listed in Supplementary Table S5. There were 168 differentially expressed miRNAs found between ES and DS groups, and 165 of them in the ES group had lower expressions than in the DS group (Supplementary Table S6). Target genes of differentially expressed miRNAs were predicted and subjected to GO and KEGG enrichments analyses, and the results indicate that predicted target genes were involved in 54 GO terms (Supplementary Table S7) and 295 pathways (Supplementary Table S8). The enriched GO terms and top 20 enriched pathways are shown in Figure 3A,B, respectively.

### 3.4. Sequencing and Analysis of mRNA and lncRNA

Groups DS and ES acquired 87,594,681 and 84,720,001 clean reads respectively, with 99.88% and 99.89% HQ clean reads, respectively. Reads unmapped to rRNA were selected and aligned to the caprine genome. In total, there were 30,688 reference isoforms, to which 80.14% (24,593) of isoforms were mapped, and 12,470 new isoforms were found.

**Figure 4:**
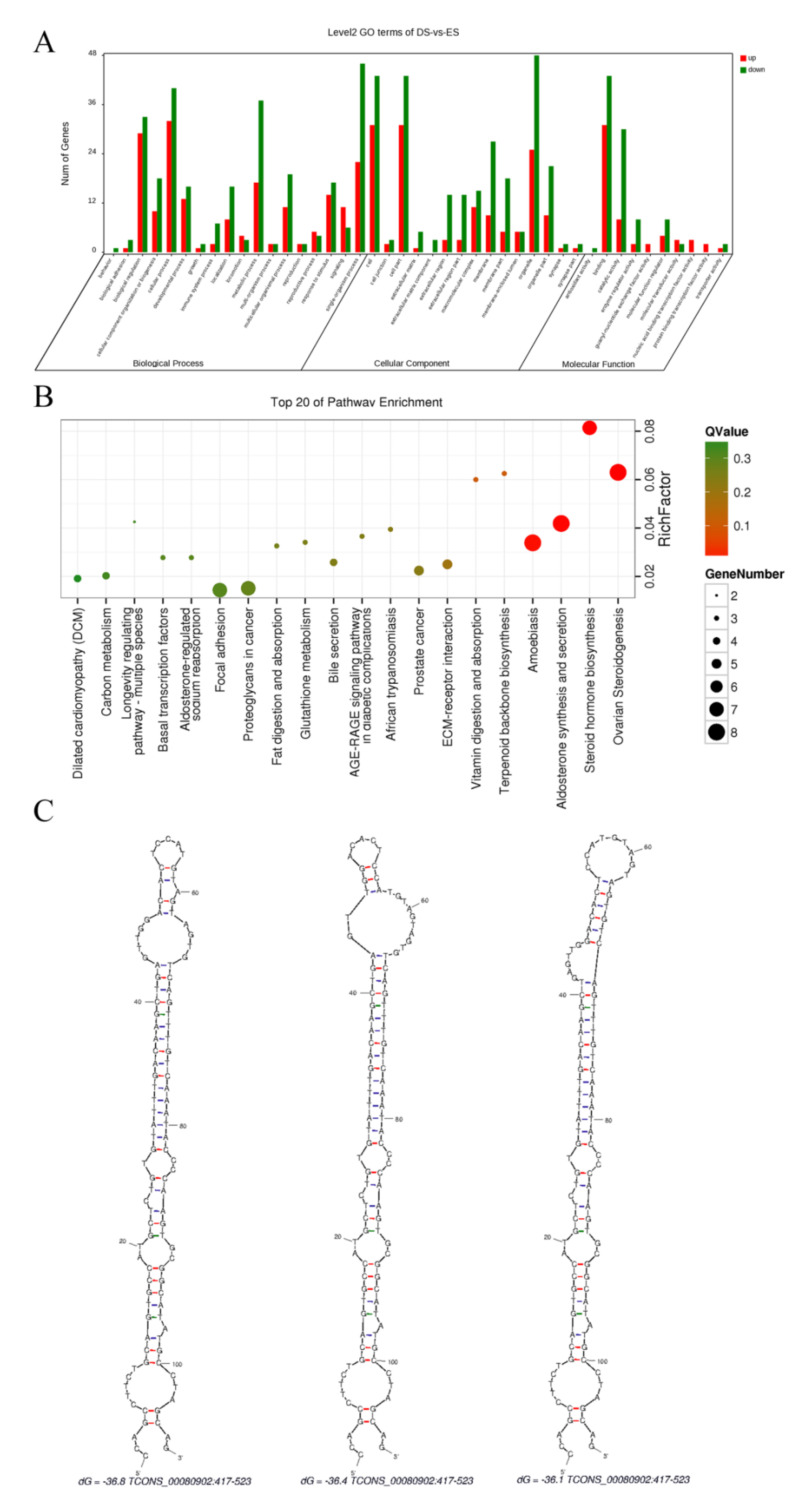
The analysis of mRNA and lncRNA. (**A**) The GO enrichment of differentially expressed mRNAs, (**B**) the KEGG pathway enrichment of differentially expressed mRNAs, and (**C**) the three predicted secondary structures of TCONS_00080902. The differentially expressed mRNAs were screened to explore the mRNA regulation in goat estrous cycle. GO enrichment (**A**) and KEGG enrichment (**B**) were performed, and 43 GO terms and 193 KEGG pathways that the 182 differentially expressed mRNAs participated in were enriched. Differentially expressed lncRNAs that could be the precursor of differentially expressed miRNAs were screened, and TCONS_00080902 was identified to be the precursor of miR-223. The secondary structures of TCONS_00080902 were predicted and displayed (**C**).

Coding transcripts of the isoforms were defined as mRNA. A total of 182 differentially expressed mRNAs were identified, of which 117 mRNAs were less expressed. *TIMP1* [30], *MMP9* [31,32], *3BHSD* [33,34], and *PTGIS* [35], which are essential for follicular and ovarian developments, were included. Enrichments were performed, and differentially expressed mRNAs were found to function in 43 GO terms and 193 pathways. The result of GO enrichment is shown in Figure 4A, and the top 20 enriched pathways are displayed in Figure 4B.

CNCI and CPC were applied to screen lncRNAs from the isoforms by coding ability. In this study, 4384 lncRNAs were found, among which 39 lncRNAs were downregulated and 2 lncRNAs were upregulated in the ES group. To explore the functions of identified lncRNAs, target genes of all lncRNAs in cis (Supplementary Table S9) and trans (Supplementary Table S10) were predicted. However, no potential target relationship between differentially expressed lncRNAs and mRNAs was found. We then analyzed all lncRNAs to predict lncRNAs that might be precursors of miRNAs (Supplementary Table S11), where one of the lncRNAs differentially expressed between the ES and DS groups, TCONS_00080902, was found to be a possible precursor of one of the differentially expressed miRNAs, miR-223. The three predicted secondary structures of TCONS_00080902 are shown in Figure 4C.

### 3.5. Prediction of circRNA-miRNA-mRNA and miRNA-lncRNA Functional Regulatory Networks

**Figure 5:**
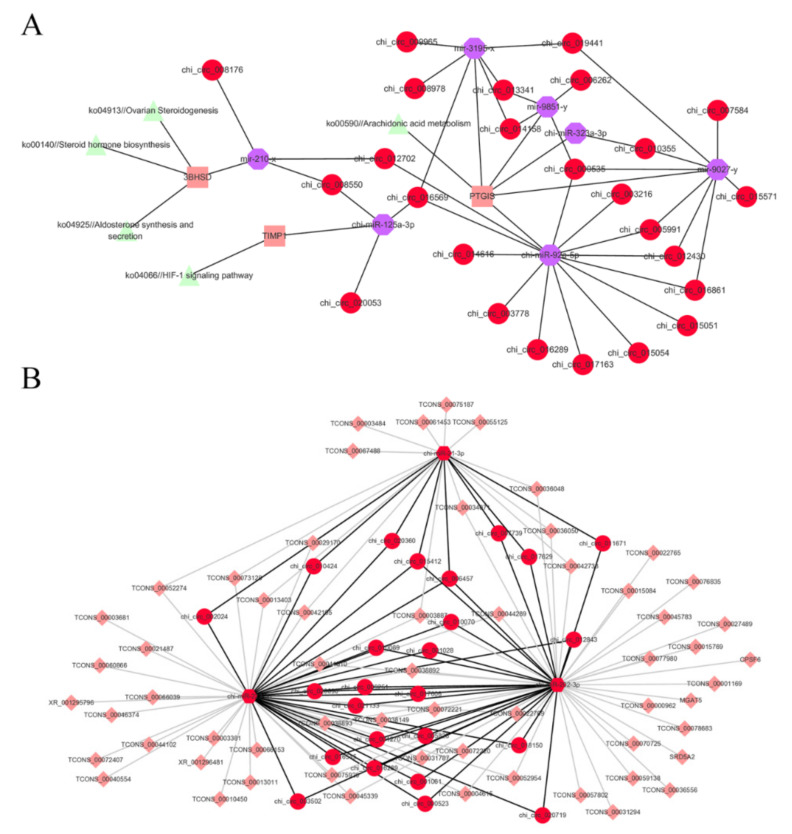
Predicted competing endogenous RNA (ceRNA) networks. (**A**) Predicted interaction between *TIMP1*, *3BHSD,* or *PTGIS* and differentially expressed miRNAs as well as circRNAs. The differentially expressed miRNAs that were predicted to target *TIMP1*, *3BHSD,* or *PTGIS* and differentially expressed circRNAs that could be miRNA sponges were screened to build a circRNA-miRNA-mRNA network.TTIMP1, 3BHSD and PTGIS were involved in the network. (**B**) Predicted target mRNAs/lncRNAs and circRNA sponges of miR-21-3p, miR-202-3p, and miR-223-3p. The circRNAs that could potentially sponge more than two of miR-21-3p, miR-202-3p, and miR-223-3p were screened to display. The predicted target mRNAs/lncRNAs were screened, and the most prominent 25 mRNAs/lncRNAs were shown in the network.

This study provides information of all predicted binding possibilities of differentially expressed miRNAs to differentially expressed circRNAs/mRNAs/lncRNAs (Supplementary Table S12). The circRNA-miRNA-mRNA network involving *TIMP1*, *3BHSD,* and *PTGIS* was explored (Supplementary Table S13). We searched for their upstream miRNAs in the differentially expressed miRNA library; besides, differentially expressed circRNAs that have potential to be the miRNAs sponges were filtered. Then, the network centering on *TIMP1*, *3BHSD,* and *PTGIS* was constructed (Figure 5A). It can be seen that *TIMP1* participates in the HIF-1 signaling pathway (ko04066), *3BHSD* participates in Aldosterone synthesis and secretion (ko04925), Ovarian Steroidogenesis (ko04913), and Steroid hormone biosynthesis (ko00140), and *PTGIS* participates in Arachidonic acid metabolism (ko00590), which are important in follicular and ovarian developments [36,37,38,39]. It is gratifying that differentially expressed miRNAs potentially targeting *TIMP1*, *3BHSD,* and *PTGIS* were screened, while these miRNAs were potentially sponged by lots of circRNAs; for clear presentation, only parts of prominent circRNAs were selected and shown in Figure 5A. This network provides the possible pathways that *TIMP1*, *3BHSD,* and *PTGIS* are involved in when DS turns to ES.

Moreover, miR-21b-3p, miR-202-5p, and miR-223-3p were selected due to their essential roles in follicular and ovarian developments [40,41,42] to analyze their target relationship with differentially expressed mRNAs/lncRNAs and sponge relationship with differentially expressed circRNAs (Supplementary Table S14). CircRNAs that potentially sponge two or more of miR-21b-3p, miR-202-5p, and miR-223-3p are shown in Figure 5B.

## 4. Discussion

In this study, databases of goat ovary mRNA and non-coding RNAs, including miRNA, circRNA, and lncRNA, were acquired, and their expressions were compared between estrus stage (ES) and diestrus stage (DS) groups. Then, differentially expressed miRNAs, circRNAs, lncRNAs, and mRNAs were screened. Abundant expression of non-coding RNAs and mRNA illustrates the subtle regulation in ovary to keep homeostasis. The circRNA-miRNA-mRNA/lncRNA and lncRNA-miRNA/mRNA networks that might be involved in the regulation of estrous cycle were predicted based on the differential expressions between ES and DS groups. Among them, the circRNA-miRNA-mRNA network that *matrix metallopeptidase 9* (*MMP9*), *tissue inhibitors of metalloproteinases* (*TIMP1*), *3β-Hydroxysteroid dehydrogenase* (*3BHSD*), and *Prostaglandin I2 Synthase* (*PTGIS*) are involved in, and the circRNA-miRNA-mRNA/lncRNA network that miR-21-3p, miR-202-3p, and miR-223-3p participated in, were extracted and displayed in Figure 5. The significant regulatory role of *MMP9* [30,31,32], *TIMP1* [30,31,32], *3BHSD* [33], *PTGIS* [43,44], miR-21-3p [45], miR-202-3p [46], and miR-223-3p [47,48] had been described in previous studies, therefore, we extracted the networks that center on them to show key potential regulation pathways of estrous cycle.

Our study established miRNA, circRNA, lncRNA, and mRNA databases of goat ovaries in ES and DS groups and analyzed the differentially expressed ones for circRNA-miRNA-mRNA/lncRNA and lncRNA-miRNA/mRNA networks that participate in estrous cycle. To make the networks concise, on the one hand, among the differentially expressed mRNAs, *MMP9*, *TIMP1*, *3BHSD,* and *PTGIS* were selected to screen the differentially expressed miRNAs and circRNAs that potentially regulated their expressions, constructing circRNA-miRNA-mRNA networks. MMP9 is one of the matrix metalloproteinases participating in extracellular matrix deconstruction, while TIMP1 is one of their endogenous tissue inhibitors [31]. The equilibrium between MMPs and TIMPs is required for extracellular matrix remodeling during ovarian folliculogenesis [30,31,32]. 3BHSD is an enzyme involved in the synthesis of progesterone and testosterone [33], which plays an essential role in estrous cycle. PTGIS is a monooxygenase that catalyzes steroids synthesis and converts prostaglandin precursor into prostaglandin I2, taking a critical role in reproductive processes [43,44]. Among differentially expressed miRNAs, we found seven miRNAs that might target *TIMP1*, *3BHSD,* or *PTGIS,* but no miRNA potentially targeted *MMP9*. Additionally, differentially expressed circRNAs that might bind with the seven miRNAs were screened. Finally, the circRNA-miRNA-mRNA network centered on *TIMP1*, *3BHSD,* and *PTGIS* was constructed.

On the other hand, miR-21-3p, miR-202-3p, and miR-223-3p were picked out to extract their circRNA sponges and target mRNAs/lncRNAs for their intense relation to women’s reproduction: miR-21-3p is associated with poor ovarian response to fertilization [45], miR-202-3p controls female fertility and regulates oogenesis [46], and miR-223-3p is involved in ovarian cancer invasion [47] and PCOS [48]. The predicted target mRNAs/lncRNAs and circRNA sponges of miR-21-3p, miR-202-3p, and miR-223-3p in the differentially expressed database were screened in this study to build the circRNA-miRNA-mRNA/lncRNA network focused on miR-21-3p, miR-202-3p, and miR-223-3p, laying a foundation for further exploration on pathways regulating estrous cycle. Furthermore, the structure of lncRNAs was analyzed and the lncRNAs that might be miRNA precursors were screened, which is helpful to figure out the possible path of miRNA formation. The databases and the two constructed networks would be a comprehensive reference for the regulation of estrous cycle in goat reproduction. For seasonally polyestrous domestic animals, it is important to manipulate the estrous cycle as the demand of consumers for goat products is all throughout the year. It would be helpful to study the regulation of estrous cycle to break the limitation of environmental factors such as photoperiod, season, and climate. In humans, the abnormal estrous cycle is related to various diseases, like ovarian carcinoma [6], anxiety [7,8], and epilepsy [9], which pose a threat to reproduction and the quality of life as well.

Collectively, our data provided the miRNA, circRNA, lncRNA, and mRNA database of goat ovary and each differentially expressed profile between ES and DS, and constructed networks among differentially expressed miRNAs, circRNAs, lncRNAs, and mRNAs, shedding light on the regulation of goat estrous cycle and the treatment of estrous cycle-related diseases.

## Data Availability

All data in the manuscript is available through the responsible corresponding author.

## Supplementary Materials

The following are available online at https://www.mdpi.com/article/10.3390/biology10060464/s1, Table S1: The information of identified circRNAs, including their source gene ID, chromosome and genomic location, length, and annotation type. Table S2: Enriched GO terms of circRNA source genes. Table S3: Enriched KEGG pathways of circRNA source genes. Table S4: The predicted interaction between all acquired circRNAs and existed miRNAs. Table S5: The sequence and expression of identified miRNAs. Table S6: The list of differentially expressed miRNA between ES and DS groups. Table S7: GO enrichment of predicted target genes of differentially expressed miRNAs. Table S8: KEGG pathway enrichment of predicted target genes of differentially expressed miRNAs. Table S9: Predicted target genes of all identified lncRNAs in cis. Table S10: Predicted target genes of all identified lncRNAs in trans. Table S11: Predicted lncRNAs that might be precursors of miRNAs. Table S12: All binding possibilities of differentially expressed miRNAs to differentially expressed circRNAs/mRNAs/lncRNAs. Table S13: The circRNA-miRNA-mRNA networks involving TIMP1, 3BHSD, and PTGIS. Table S14: Predicted interaction between differentially expressed mRNAs/lncRNAs/circRNAs and miR-21b-3p, miR-202-5p, and miR-223-3p.

## Abbreviations

ES	estrus stage
DS	diestrus stage
PCOS	polycystic ovary syndrome
miRNA	microRNA
circRNA	circular RNA
lncRNA	long non-coding RNA
mRNA	messenger RNA
MMP9	matrix metallopeptidase 9
TIMP1	tissue inhibitors of metalloproteinases
3BHSD	3β-Hydroxysteroid dehydrogenase
PTGIS	Prostaglandin I2 Synthase
HQ	high quality
